# Understanding the Security and Privacy Concerns About the Use of Identifiable Health Data in the Context of the COVID-19 Pandemic: Survey Study of Public Attitudes Toward COVID-19 and Data-Sharing

**DOI:** 10.2196/29337

**Published:** 2022-07-07

**Authors:** Charlotte Summers, Frances Griffiths, Jonathan Cave, Arjun Panesar

**Affiliations:** 1 DDM Health Coventry United Kingdom; 2 Warwick Medical School University of Warwick Coventry United Kingdom; 3 Department of Economics University of Warwick Coventry United Kingdom; 4 Data Ethics Group The Alan Turing Institute London United Kingdom

**Keywords:** COVID-19, data, data ethics, privacy, sharing, ethics, attitude, perception, data sharing, survey, understanding, security, health data, willingness

## Abstract

**Background:**

The COVID-19 pandemic increased the availability and use of population and individual health data to optimize tracking and analysis of the spread of the virus. Many health care services have had to rapidly digitalize in order to maintain the continuity of care provision. Data collection and dissemination have provided critical support for defending against the spread of the virus since the beginning of the pandemic; however, little is known about public perceptions of and attitudes toward the use, privacy, and security of data.

**Objective:**

The goal of this study is to better understand people’s willingness to share data in the context of the COVID-19 pandemic.

**Methods:**

A web-based survey was conducted on individuals’ use of and attitudes toward health data for individuals aged 18 years and older, and in particular, with a reported diagnosis of a chronic health condition placing them at the highest risk of severe COVID-19.

**Results:**

In total, 4764 individuals responded to this web-based survey, of whom 4674 (98.1%) reported a medical diagnosis of at least 1 health condition (3 per person on average), with type 2 diabetes (n=2974, 62.7%), hypertension (n=2147, 45.2%), and type 1 diabetes (n=1299, 27.4%) being most prominent in our sample. In general, more people are comfortable with sharing anonymized data than personally identifiable data. People reported feeling comfortable sharing data that were able to benefit others; 66% (3121 respondents) would share personal identifiable data if its primary purpose was deemed beneficial for the health of others. Almost two-thirds (n=3026; 63.9%) would consent to sharing personal, sensitive health data with government or health authority organizations. Conversely, over a quarter of respondents (n=1297, 27.8%) stated that they did not trust any organization to protect their data, and 54% (n=2528) of them reported concerns about the implications of sharing personal information. Almost two-thirds (n=3054, 65%) of respondents were concerned about the provisions of appropriate legislation that seeks to prevent data misuse and hold organizations accountable in the case of data misuse.

**Conclusions:**

Although our survey focused mainly on the views of those living with chronic health conditions, the results indicate that data sensitivity is highly contextual. More people are more comfortable with sharing anonymized data rather than personally identifiable data. Willingness to share data also depended on the receiving body, highlighting trust as a key theme, in particular who may have access to shared personal health data and how they may be used in the future. The nascency of legal guidance in this area suggests a need for humanitarian guidelines for data responsibility during disaster relief operations such as pandemics and for involving the public in their development.

## Introduction

The World Health Organization declared the COVID-19 outbreak a public health emergency on January 30, 2020; after 6 weeks, it was categorized as a pandemic [1]. Certain groups of people are particularly likely to have serious or severe symptoms of COVID-19 [2]. Preliminary data suggest that people with obesity are at an increased risk of severe COVID-19 [3]. Type 2 diabetes mellitus and hypertension are the most common comorbidities in patients with COVID-19 [4]. According to several reports, including those from the Centers for Disease Control and Prevention, patients with type 2 diabetes are at a greater risk of death than those without type 2 diabetes [5].

Digital health technologies are being used in the fight against COVID-19 [6]. Global health care systems have seen an influx in the incidence of the same novel condition, and the contagious nature of the condition has driven the shift to remote medicine. Many health care pathways have been rapidly digitalized with face-to-face services seeing a drop in usage [7]. This has increased the collection, sharing, and use of data in digital form. Technology is used for remote monitoring, general practitioner consultations, providing structured education, and tracking the spread of disease. As well as the technologies themselves, the data they generate are also useful [8].

Timely, secure, and reliable data access and sharing are critical to understanding COVID-19, controlling its spread, improving the effectiveness and acceptance of government policies, and fostering global cooperation in the race to develop and distribute effective therapies and vaccines. During the COVID-19 pandemic, data are being rapidly shared to understand the location of infections, confirmed cases, recoveries, and deaths. The main data points of interest for this are geolocation and biometric data, both of which are available from users’ mobile devices. However, there are serious concerns regarding the objectivity and accuracy of these data, and their utility has been compromised by inconsistent collection and definitions. This, in turn, feeds back into individuals’ trust in the collecting organizations and in the extent to which their shared data will actually be used to help others, and this needs to be matched by the trustworthiness of those organizations.

During an unprecedented time, some digital responses to the crisis have precipitated novel data governance and privacy challenges [9]. Governments are taking extraordinary measures to track, trace, and contain the spread of COVID-19 by transitioning to digital technologies and advanced analytics to collect, process, and share data for effective frontline responses. Government-mandated apps are bringing the fight against COVID-19 onto users’ devices and have generally adopted pragmatic and contextualized approaches, but they have prompted concerns about security and privacy and the control and use of data beyond the pandemic [10]. There is a trade-off between effectiveness and privacy, centralized and decentralized implementations, and the links to trace and isolate policies.

While the exceptional measures implemented in some countries may prove effective in limiting the spread of the virus, some have provoked controversy in terms of privacy and other fundamental rights, particularly when they lack transparency and public consultation [11]. In South Korea, the specificity of publicly available anonymized data raised privacy concerns when some researchers found that data trails were so detailed that individuals could be identified [12]. In Italy, the Department of Prevention released specific guidelines on the application of the European Union’s and national data privacy rules in the context of the COVID-19 pandemic [13]. Similarly, the United Kingdom’s Information Commissioner's Office, an independent authority set up to uphold information rights in public interest, confirmed that there would be no regulatory action taken against organizations that fail to meet the data protection standards if noncompliance results from the COVID-19 pandemic [14,15]. In China, new arrivals to the country are tested for COVID-19, instructed to download a government-mandated app, and wear a wristband that is linked to the app to monitor movement with a technology similar to that used in Singapore [16,17]. The United Kingdom’s Track and Trace app was the center of a debate on centralization of data [18]. On May 5, 2020, the Government revealed its first attempt at a contact-tracing app, but 6 weeks later admitted that the app was flawed and it would switch to a more privacy-preserving model devised by Apple and Google [19,20]. Transparency is a key theme. One of the most common misconceptions about the United Kingdom’s Track and Trace app was that it could allow users to specifically identify and map COVID-19 cases among their contacts and in their vicinity [21].

This study seeks to understand the opinions of British people with long-term health conditions on the themes of data privacy and security, data ethics, and data misuse and to assess the possible trade-offs in data utilization to manage a crisis such as the COVID-19 pandemic [22]. It is important to understand the concerns of people with long-term health conditions such as type 2 diabetes and hypertension as these conditions have been shown to be key risk factors in the progression and prognosis of COVID‐19 [23,24].

## Methods

### Study Design and Setting

A web-based survey study was conducted with a mixed methods design conforming to the checklist for reporting results of internet electronic surveys [25]. An email invitation to participate, which included a weblink to the survey, was sent to 11,213 people who had consented to be contacted for research opportunities.

Quantitative information (closed and multiple-choice questions) was collected on four topics: (1) demographic characteristics, (2) COVID-19 symptoms and clinical diagnoses, (3) sharing and privacy of pre– and post–COVID-19 health data, and (4) COVID-19 lockdown behaviors. Responses from the final topic are not included in this analysis.

The survey contained 31 questions: 26 closed questions, 1 open question, and 4 demographic questions. Questions on sharing and privacy of pre– and post–COVID-19 health data were answered on a 5-point Likert scale with responses ranging from *strongly disagree* to *strongly agree* or from *not concerned at all* to *very concerned*.

### Participants

People aged ≥18 years who had joined the Diabetes.co.uk community were surveyed. The survey commenced with 1 screening question: “Do you consent to take part in the study?” Respondents who consented went on to complete the survey.

### Procedure

Data collection occurred between July 6 and August 31, 2020. The survey was administered through the Jisc Online Surveys software and comprised closed, open, and multiple-choice questions. The survey was designed to elicit individual responses to questions about retrospective data use and privacy prior to the COVID-19 pandemic and prospective use during the COVID-19 pandemic.

It is intended to have multiple windows of data collection for several reasons: people’s recollections of pre–COVID-19 attitudes may be unreliable, and changes in the course, apparent seriousness, and confidence in scientific understanding of the pandemic will have evolved.

The type and wording of each question was composed by the research team. The order of questions was not randomized. The survey followed a predetermined logic where contingent questions were included or automatically skipped on the basis of responses. Qualitative data were collected with 1 open question exploring what respondents would like to see happening: “What would you like to see happen to improve the COVID-19 situation?” (question 30).

### Analysis

We exported all data from Jisc and conducted data analysis using SPSS (version 22; IBM Corp). We conducted descriptive data analyses of sample distributions and characteristics. Pearson *r* correlation coefficients were used to determine the relation between prior data-sharing behavior and attitudes toward data-sharing activity in the context of the COVID-19 pandemic. The data from the open question were read through and then categorized into themes.

### Ethical Considerations

Ethics approval was obtained from the Human Research Ethics Committee of the University of Warwick (BSREC 144/19-20). Web-based informed consent was required before the survey could be accessed.

## Results

### Survey Respondents

Of 11,213 people emailed, 10,705 clicked through to the survey; in total, 4764 gave their consent and began the survey. As indicated in Table 1, all of them completed the survey and were included in the analysis. All respondents were located in the United Kingdom. In total, 2287 (48.0%) respondents were male and 3083 (64.8%) were aged between 55 and 74 years. A total of 115 (2.8%) respondents reported having been clinically diagnosed with COVID-19. The majority of patients (n=4674, 98.1%) reported a prior clinical diagnosis of at least one health condition (on average 3 per person). There was a high prevalence of individuals living with type 2 diabetes (n=2974, 62.7%), hypertension (n=2147, 45.2%), type 1 diabetes (n=1299, 27.4)*,* obesity (n=892, 18.8%), and depression (n=871, 18.3%). Respondent demographics are shown in Table 1.

**Table 1:** Respondent demographics (N=4764).

Characteristics		Respondents, n (%)	
**Gender**			
	Male		2287 (48.0)
	Female		2435 (51.1)
	Prefer not to say		42 (0.9)
**Age (years)**			
	18 to 24		23 (0.5)
	25 to 34		104 (2.2)
	35 to 44		298 (6.3)
	45 to 54		839 (17.6)
	55 to 64		1550 (32.6)
	65 to 74		1533 (32.2)
	75 or older		410 (8.6)
	Prefer not to say		7 (0.1)
**Health conditions^a^**			
	Type 2 diabetes		2974 (62.7)
	Hypertension		2147 (45.2)
	Type 1 diabetes		1299 (27.4)
	Arthritis		1002 (21.1)
	Obesity		892 (18.8)
	Depression		871 (18.3)
**Employment^b^**			
	Full-time employment		1205 (24.9)
	Part-time employment		571 (11.8)
	Retired		2298 (47.5)
	Student		39 (0.8)
	Unemployment		464 (9.6)
	Furloughed		188 (3.9)
	Volunteering in my community (National Health Service, key services)		69 (1.4)
**Ethnicity**			
	Indian or Pakistani		69 (1.4)
	Black, British African, or Caribbean		46 (1.0)
	Middle Eastern		5 (0.1)
	Mixed groups		27 (0.6)
	White		4,434 (93.1)
	Other		44 (0.9)
	Chinese, Japanese, or East Asian		7 (0.1)
	Prefer not to say		132 (2.8)

^a^Frequently occurring health conditions selected.

^b^Respondents selected multiple statuses; for example, full-time employed and furloughed.

### COVID-19 Symptoms and Clinical Diagnosis

Of the 4764 respondents who completed the survey, 494 stated they had had symptoms of COVID-19. The most common symptoms were the following: a continuous cough (n=467, 94%), fever (n=325, 65.7%), difficulty breathing (n=384, 77.7%), and loss of taste (n=324, 65.5%). In total, 384 (77.7%) respondents reported another symptom, predominantly fatigue (16.4%).

Of those reporting symptoms, 111 (22.5%) reported a clinical diagnosis of COVID-19. Of these respondents, 73 (63.5%) reported that their symptoms were severe or very severe. In total, 26 (22.6%) respondents reported that their symptoms were not severe at all. All respondents who reported a clinical diagnosis of COVID-19 reported at least one symptom, including loss of smell or taste (63.1%), fever (62.2%), difficulty breathing (61.3%), or continuous cough (53.2%).

A total of 131 (2.8%) respondents reported that a household member had been tested and was clinically diagnosed with COVID-19.

### Sharing and Privacy of Pre–COVID-19 Health Data

Prior to the COVID-19 pandemic, almost half of the respondents (n=2313, 49.2%) agreed or strongly agreed that they often consented to anonymized sharing of their private health data, while only 608 (13%) respondents often consented to sharing of private health data without anonymization. Two-thirds of respondents (n=3113, 66.7%) disagreed or strongly disagreed with sharing their private health data without anonymization. Similarly, 3121 (66.3%) respondents would share their data if it keeps other people healthy; 3026 (63.9%) respondents agreed or strongly agreed to sharing private health data with the government or health authority; 1911 (40.7%) respondents agreed or strongly agreed to share their private health data with services that provide health services to the National Health Service (NHS) such as the Low Carb Program and PushDoctor. Only 232 (5%) participants agreed or strongly agreed to share private health data with social media platforms. Over a quarter of respondents (n=1297, 27.8%) agreed or strongly agreed that they did not trust *any* organization to protect their private health data. Just under a quarter of respondents (n=1094, 23.5%) agreed or strongly agreed that they were not concerned by the implications of sharing private health data. General health data–sharing responses are shown in Table 2. Respondents who reported that they felt “neutral” in response to the statements were excluded.

**Table 2:** General health data sharing responses.

Question	Disagree or strongly disagree, n (%)	Agree or strongly agree, n (%)
I often consent to share my private health data with any organisation as long as it is anonymised	1273 (27.1)	2313 (49.2)
I often consent to share my private health data to any organisation without anonymisation	3113 (66.7)	599 (13)
I am not concerned about the implications of sharing my private health data	2528 (54.2)	1094 (23.5)
I don't trust any organisation to protect my private health data	1541 (33.8)	1297 (27.8)
I'm happy to share my private health data if it helps keep other people healthy	526 (11.2)	3121 (66.3)

### Sharing and Privacy of Post–COVID-19 Health Data

Over half (n=3026, 63.9%) agreed or strongly agreed to share their private data with the government or health authority if asked; 1911 (40.7%) respondents would happily consent to share their private data with services that provide health services to the NHS such as the Low Carb Program and PushDoctor, if asked. Only 232 (5%) participants agreed or strongly agreed that they would consent to sharing private data with social media if asked.

Almost half of respondents (n=2228, 47.1%) were concerned or very concerned about who would have access to their personal health data in the context of the COVID-19 pandemic and 2310 (49.1%) respondents were concerned or very concerned about how their personal health data may be used in the future. Almost two-thirds of respondents (n=3054, 65%) were concerned or very concerned around the legislation of data misuse.

Just over a third of respondents (n=1563, 33.4%) would consent to share their private data with any organization if it was providing essential COVID-19 support services such as the supermarkets, pharmacies, and banks. Responses toward the use of post–COVID-19 patient data is shown in Table 3, along with the sentiment toward the use of patient data in the context of the COVID-19 pandemic, and Table 4 shows the sentiment toward future use or misuse of data collected and used under the provisions of the COVID-19 pandemic.

**Table 3:** Responses toward the use of post–COVID-19 patient data.

Question	Disagree or strongly disagree	Agree or strongly agree
I would happily consent to share my private health data with the government or health authority	728 (15.4)	3026 (63.9)
I would happily consent to share my private health data with social media e.g Twitter, Facebook, Google	4023 (85.9)	232 (5)
I would happily consent to share my private health data with services that provide health services to the NHS^a^ such as Low Carb Program, PushDoctor, Babylon Health	1351 (18.8)	1911 (40.7)

^a^NHS: National Health Service.

**Table 4:** Sentiment toward future use or misuse of data collected and used under the provisions of the COVID-19 pandemic.

Question	Not concerned at all	Concerned or very concerned
In light of COVID-19, how concerned are you about who would have access to your personal health data?	1138 (14)	2228 (47.1)
How concerned are you about how your personal health data may be used in the future?	1162 (24.7)	2310 (49.1)
How concerned are you around the legislation of data misuse?	644 (13.6)	3054 (65)

### Prior Willingness to Share Data

Correlations between retrospective data-sharing that happened in the context of generalized concerns and attitude changes associated with the course of the pandemic were determined. Changes were not linked to any specific studies, policies, or measures. There were strong correlations in the attitudes of people exhibiting high levels of concern about future uses of shared data and concerns about access (*r*_4685_=0.816; *P*<.001). There was a strong correlation between people exhibiting attitudes of concern that firmer legislation for data misuse is needed and concerns about future repurposing and reuse of personal health data collected during the COVID-19 pandemic (*r*_4663_=0.636; *P*<.001). The Pearson *r* correlation coefficient as a normalized measure of the strength of a possible linear correlation, lying between –1 and +1. The Pearson *r* correlation coefficient measures nonlinear correlations (eg, when extreme views are highly correlated but more moderate ones are more independent).

Respondents agreed to share their personal data with roughly the same parties prior to the COVID-19 pandemic and within the context of the COVID-19 pandemic; governments and health authorities (*r*_4710_=0.762; *P*<.001), health service providers such as the Low Carb Program and PushDoctor (*r*_4662_=0.783; *P*<.001), and social media platforms such as Twitter, Facebook, and Google (*r*_4662_=0.736; *P*<.001).

### COVID-19 News and Information

Of the 4764 respondents, 2666 (56.1%) were concerned that they may be receiving misinformation about COVID-19 from trusted sources, 1079 (22.7%) were not concerned (genuinely unconcerned and those who feel that they are in control of the consumption of news and information), and 1006 (21.2%) had never considered it.

In total, 4237 responded to the open-ended question of what they would like to see happening to improve the COVID-19 situation. The majority of respondents shared a single response: 1348 (31.8%) stated they would like to see a reliable vaccine and treatment, 884 (20.8%) stated they would like to see balanced information from the government, and 485 (11.4%) wanted to see stricter measures to prevent the transmission of COVID-19.

## Discussion

### Principal Findings

Our study provides insights into public perception and attitudes toward the use of identifiable health data in the context of the COVID-19 pandemic; in particular, the perspectives of those living with chronic, long-term health conditions, with an average of 4 health conditions reported per respondent.

Our study suggests that data sensitivity is highly contextual. A significant proportion of people felt that their own attitudes have shifted as a result of the COVID-19 pandemic. More people reported being comfortable with sharing private health data with any organization during rather than before the COVID-19 pandemic. In order, people appear to trust their data with the government, health organizations, and social media. There is significant distrust of private health data use by social media organizations (eg, Twitter, Facebook, and Google) even though social media is used as a channel for communication by people caught up in crises such as emergency relief operations after earthquakes, tsunamis, and typhoons; where it provides a trusted and highly salient source of information about what is happening and what to do [26,27]. This is surprising as although users worldwide report that privacy and use of personal data are important issues, most rarely make an effort actively to protect these data and often even give them away voluntarily on social media where even innocuous data can reveal sensitive health information when suitably processed [28,29]. People treat data revelation and sharing differently depending on the perceived sensitivity of the data, and the sensitivity attached to different types of data is neither stable nor uniform.

When examining the correlations between retrospective views of data-sharing behavior and comfort regarding data-sharing in the context of the COVID-19 pandemic, individuals were comfortable (or not) in sharing personal data with the same organizations prior to and during the COVID-19 pandemic, suggesting that COVID-19 has not drastically shifted people’s willingness to share or withhold their personal data. This may be because attitudes have shifted both retrospectively and prospectively and also depends on whether people accurately remember and report their past views and actions. One of the strongest correlations observed in the analysis was between high levels of concern about the requirement for stronger legislation protecting individuals from data misuse and future repurposing and reuse. This highlights the need for improved communication, transparency, and potentially stronger regulation on how such data may be repurposed in the future, who will be accountable for inappropriate use of data, and a commitment to cease or reverse exceptional uses of data when the crisis is over. Individuals’ data rights are protected by law in regulation such as General Data Protection Regulation 2018 in Europe and Health Insurance Portability and Accountability Act in the United States, which make clear the scope, purpose, and time limitations of data usage [30,31]. Concerns may therefore reflect ignorance of existing rules, doubts over enforcement, or a belief that current legislation does not go far enough (for instance, in the requirement of erasure after 3 years rather than a shorter time duration).

A key theme emerging from the literature that was confirmed in this study is the importance of trust [32-34]. Over a quarter of respondents stated they did not trust any organization to protect their data, over half reported concern about the implications of sharing personal information, and almost two-thirds were concerned about data misuse regulation not being strict enough. When asked during the pandemic (the United Kingdom’s first wave), almost half of respondents were concerned about who would have access to their personal health data and a similar number were concerned about how their personal health data might be used in the future. This is consistent with prior research suggesting that public involvement in data policy is crucial to bolstering trust and provides support for legislation that is more enforceable [35]. Attitudes may have been perturbed by news stories relating to cybersecurity and privacy and by policy announcements (eg, around Huawei, the Online Harms Bill, etc) [36,37].

Although there are no directly comparable studies, the results from this study complement prior research on public perceptions about COVID‐19 and data-sharing. Data privacy and protection are important concepts [38]. Data policy tends to address human concerns about privacy by making rules about data protection; however, this can lead to category errors since data protection can undermine privacy.

Willingness to share anonymized personal health information varies depending on the degree to which the receiving body is trusted and the uses to which the data will be put [39,40]. The more commercial the objectives of the receiving institution appear, the less respondents are willing to share their personal health information. This in turn suggests that anonymization’s disadvantages (in terms of confirming data and correlating shared with other data) might be offset by better (wider, deeper, and more accurate) sampling leading to greater validity of results. Further evidence comes from the interaction (or correlation) between these attitudinal responses and other characteristics, meaning that nonanonymized collection might lead to biased results.

Virus tracking apps are used at scale by governments; however, concerns about transparency, privacy, and morality remain [41,42]. There has been substantial research into the challenges involved in the digital response to the COVID-19 pandemic and proposed methodologies for the ethical design and use of digital public health tools [43,44]. Clear and effective data ethics is both a moral and a practical obligation. The nascency of legal guidance in this area combining ethics, law, and humanitarian impulses suggests the requirement for humanitarian guidelines for data responsibility during global crises such as pandemics. Therefore, rather than recalibrating the expectations of people with regard to their own privacy, the requirements for the use of data should be broader and more comprehensive as ethically collected big data could prove to be extremely useful in the prediction, monitoring, and mitigation of pandemics such as COVID-19 [45].

### Strengths and Limitations

Despite the importance of the findings reported here, it is important to note that this study had several limitations. Conducting this study via a web-based survey carries a risk of response bias, simply because the respondents are likely to be more technology-savvy than the general population. However, the population studied (those with chronic health conditions) is of interest as these participants have a degree of awareness and the ability to self-manage their condition that is not (yet) typical of the population at large, and this sheds light on how policies that raise awareness may lead to greater effectiveness in terms of uptake of technical solutions and effectiveness of public health advice and other policies.

Participants were asked to rate retrospectively their perceptions of data sharing prior to the COVID-19 pandemic; these ratings may be inaccurate owing to faulty memories and response bias. There is some ambiguity between what people (now) thought they would have done had they been asked and how they responded to actual requests for consent to data-sharing. In particular, one could disagree with a statement like “I often consent to share my private health data to any organisation without anonymisation” simply because one rarely recalls being asked to share (even without a principled objection to such sharing should the occasion arise). In addition, the phraseology of the questionnaire refers to data that have either been anonymized or are identifiable. This dichotomous representation leaves out pseudonymized data. The participants were not educated about the concepts of anonymization prior to answering the questions potentially allowing ambiguity of the terminology to cause a strong bias in the response behavior.

In addition, the sample is concentrated on people with diabetes and those with other diagnosed health conditions, rather than the general population. This is a strength as well as a weakness as it focuses on a population with particular circumstances and perspectives and one that may be more representative of a post–COVID-19 population that has been sensitized to a continuing health concern than the current population. This in turn means that a comparison of these findings with a similar survey of the general population can shed light on the potential impact of awareness-raising policies.

Another strength of the study is the high number of respondents who completed the questionnaire. In total, 4764 people participated in the study. This provides a unique insight into the views of a population deemed as being at the highest risk of severe disease and mortality related to COVID-19 [46]. There was a skew in the representation of the demographic distribution of individuals in the nationwide population of people living with chronic health conditions, since White people were overrepresented in our sample (n=4434, 93.4%) but not overrepresented among those with diabetes more generally. While our survey focused on those with diabetes, the results provide novel insight into concepts crucial for societal trust in data use and sharing initiatives.

While the study design did not allow us to ascertain whether technology use itself was correlated with higher acceptance of data-sharing, such an analysis is possible and will be an important topic for future research.

The study’s findings suggest potential targets for further study and possible considerations for policy makers. There are two main implications: storing and processing data in pseudonymized form and emphasizing the use of synthetic data (generated from models estimated from real data but not involving any actual or identifiable human beings).

Understanding attitudes toward data sensitivities and trust can contribute to developing policies, improving transparency, and increasing the trust, speed, focus, and effectiveness of epidemic responses. Future practice should emphasize transparent data-sharing and privacy initiatives, while research should evaluate whether this does indeed lead to greater levels of trust and engagement. Encouraging ethical and relevant data-sharing can provide significant epidemic intelligence and support public health emergency relief operations [47].

### Conclusions

Data sensitivity is highly contextual. More people are comfortable with sharing anonymized data than personally identifiable data. Willingness to share data also varied depending on the receiving body, highlighting trust as a key theme, who may have access to shared personal health data and how it may be used in the future. The nascency of legal guidance in this area suggests the requirement for humanitarian guidelines for data responsibility during disaster relief operations such as pandemics, and the requirement to involve the public in their development.

## Abbreviations

NHS: National Health Service
